# Association between ethnicity and emergency department visits in the last three months of life in England: a retrospective population-based study using electronic health records

**DOI:** 10.1136/bmjph-2024-001121

**Published:** 2024-10-18

**Authors:** Joanna M Davies, Javiera Leniz, Kia-Chong Chua, Lesley E Williamson, Sabrina Bajwah, Thomas Bolton, Anna E Bone, Mevhibe Hocaoglu, Julia Verne, Lorna K Fraser, Stephen Barclay, Fliss E M Murtagh, Irene J Higginson, Katherine E Sleeman, on behalf of the CVD-COVID-UK/COVID-IMPACT Consortium

**Affiliations:** 1 Department of Palliative Care, Policy and Rehabilitation, Cicely Saunders Institute, King's College London, London, UK; 2 Departamento de Salud Pública, Pontificia Universidad Católica de Chile, Santiago, Chile; 3 Centre for Implementation Science, Health Service and Population Research, Institute of Psychiatry, Psychology and Neuroscience, King's College London, London, UK; 4 Kings College Hospital NHS Foundation Trust, London, UK; 5 Health Data Research UK, British Heart Foundation Data Science Centre, London, UK; 6 Primary Care Unit, Department of Public Health and Primary Care, University of Cambridge, Cambridge, UK; 7 Wolfson Palliative Care Research Centre, Hull York Medical School, University of Hull, Hull, UK

**Keywords:** Epidemiology, Emergencies, Public Health, Epidemiologic Factors, Sociodemographic Factors

## Abstract

**Introduction:**

Emergency department (ED) visits are distressing yet common in the last months of life and many could be avoided. The association between ethnicity and ED visits in the last months of life has rarely been studied in detail and the intersection with area-based deprivation and other risk factors is not known.

**Methods:**

Population-based, retrospective cohort study, using electronic health records for adults who died from all causes in 2019 and 2020 in England.

**Results:**

Of 566 930 deaths in 2020, 356 700 (62.9%) had at least one ED visit in the last 3 months of life. Most ethnic minority groups had more ED visits than white British people and differences were larger for visits out-of-hours. After adjusting for social and clinical factors, compared with white British people, the out-of-hours visit rate for people with Bangladeshi, Pakistani and Indian ethnicities was 17% (95% CI 6% to 28%), 19% (95% CI 12% to 27%) and 14% (95% CI 6% to 22%) higher for women, and 16% (95% CI 9% to 23%), 13% (95% CI 8% to 19%) and 6% (95% CI 0% to 12%) higher for men. The rate of visits was lower in 2020 than in 2019, but differences between ethnic groups were similar. For white British people, there is a clear social gradient—those who live in more deprived areas have a higher rate of ED visits—but this is not seen for most other ethnic groups.

**Conclusion:**

People with Bangladeshi, Indian and Pakistani ethnicities have higher rates of ED visits in the last 3 months of life that are not fully explained by other social and clinical factors. This difference is driven by visits out-of-hours, which may indicate a need for better support. Future work should try to understand why some ethnic minority groups use ED more and how this relates to differences in needs, preferences and experiences.

**WHAT IS ALREADY KNOWN ON THIS TOPIC**

- Emergency department visits towards the end of life are common and can be distressing and costly, and some are avoidable through better community care.
- The association between ethnicity and emergency department visits in the last months of life has rarely been studied in detail and the intersection with other demographic and clinical factors is not known.

**WHAT THIS STUDY ADDS**

- Ethnic inequalities in emergency department visits emerge most strongly in the out-of-hours period (evenings, weekends and bank holidays).
- Most ethnic minority groups have more emergency department visits in the last 3 months of life than white British people; for people with Bangladeshi, Pakistani and Indian ethnicities, age, geography, area-based deprivation and morbidity only partially explain this association.

**HOW THIS STUDY MIGHT AFFECT RESEARCH, PRACTICE OR POLICY**

- This study highlights the importance of recognising intersecting risk factors for emergency department visits and the need to understand *why* some ethnic minority groups have a higher rate of emergency department visits in the last months of life, particularly during the out-of-hours period.

## Introduction

 The increasing demand on emergency departments has been described as an international crisis, with emergency admissions acting as a barometer for pressure on other parts of the health and social care system.^1 2^ For people in the last year of life, emergency department visits are common and increase rapidly in the months before death.^3 4^ Visits to the emergency department towards the end of life can be distressing; some are appropriate but others could be avoided through better community support.^5^,^7^ The out-of-hours period (evenings, weekends and bank holidays) can be a particular concern as patients with escalating and deteriorating symptoms need access to care around the clock, and out-of-hours care for people with advanced illness is variable and fragmented.^4 8^

People living in more deprived areas have higher rates of emergency department visits at all stages of life, including in the last year of life.^4 9^ Along with area-based deprivation, other predictors of emergency admissions towards the end of life include rurality, having more comorbidities, and having non-white ethnicity.^10^,^12^ Evidence on the association between race or ethnicity and emergency department visits in the last months of life is largely based on data from the USA, and there is very little analysis of the intersection between ethnicity, socioeconomic position, geography, and health.

In the UK, the COVID-19 pandemic exacerbated existing inequalities in health, mortality, and access to services, including for people with terminal illness.^4 13^ For example, the number and proportion of people dying at home (compared with hospitals) increased during the pandemic, but this increase was greatest for people living in less deprived areas, which may indicate inequitable access to community-based care and support.^14^ The higher mortality from COVID-19 experienced by ethnic minorities has highlighted the need to understand ethnic inequalities in health and society more broadly.^15^

This study makes use of nationally linked data resources for England^16^ to investigate the relationship between ethnicity and emergency department visits for people in the last months of life, in the context of the COVID-19 pandemic. The aim is to analyse the association between ethnicity and emergency department visits in-hours and out-of-hours in the last 3 months of life, before and during the COVID-19 pandemic and to understand how far this relationship is explained by other characteristics, including geographical factors, area-based deprivation, and morbidity.

## Materials and methods

### Study design, data sources, setting and participants

This retrospective population-based study analyses data for all adults (≥18 years old) who died in England, excluding people who were in hospital for the full duration of their final 3 months of life. The main analysis includes all deaths in 2020. The analysis investigating the effect of the COVID-19 pandemic includes all deaths between 1 July and 31 December 2020 and for the same period in 2019. This time period was selected to ensure that all the emergency visits for deaths in 2020 took place after the start of the first UK COVID-19 lockdown in March 2020.

The analysis uses five datasets, including (1) mortality records from the Office of National Statistics, linked to (2) Hospital Episode Statistics (HES) Accident and Emergency (A&E), (3) HES Admitted Patient Care, (4) primary care prescriptions and (5) General Practice Extraction Service (GPES) Data for Pandemic Planning and Research (GDPPR). We started with the mortality data; all other datasets were linked to this. The GPES GDPPR includes 98% of general practices and approximately 96% of the general population^16^; all other datasets cover the full population.

Data were accessed through NHS England’s Secure Data Environment service for England via the British Heart Foundation Data Science Centre’s CVD-COVID-UK/COVID-IMPACT Consortium.

### Study variables

The outcome is the number of emergency department visits (including visits to major A&E departments, single specialty A&E departments, walk-in centres, and minor injury units) per person in the last 3 months of life. We concentrated on the last 3 months of life because our earlier analysis revealed a steep increase in emergency department visits in the 3 months before death.^4^ Emergency department visits were classified as in-hours or out-of-hours based on the time and day of occurrence. Out-of-hours was defined as visits occurring after 18:00 and before 8:00, at weekends, and on bank holidays. Visits for the same patient with the same date and time were treated as duplicates and dropped from the analysis; otherwise, multiple visits on the same day were included.

The main exposure is ethnicity, taken from the most recent primary care record or the hospital episode statistics record, prioritising the primary care record, thus providing a more complete record than using hospital records alone.^16^ Ethnic groups were organised into the nine categories used by the Office for National Statistics^17^ with the additional separation of White British from White other. Covariates include sex and age at death; other covariates are summarised in table 1 below.

**Table 1:** Summary of covariates

Geographical variables: population density and LA(linked to the most recently recorded postcode of patient residence)	Mid-2020 population density at LSOA level as a continuous variable. There are 32 844 LSOAs in England with an average population of 1500 people or 650 households. Population density is calculated as the population estimate for each LSOA, divided by its land area in square kilometres.^36^LA; one of 317 LAs in England.
Area-based deprivation(linked to the most recently recorded postcode of patient residence)	We used an area-based measure of deprivation as a proxy for individual-level socioeconomic position. Level of ‘neighbourhood’ deprivation is based on the Index of Multiple Deprivation for England (2019), which ranks LSOAs in England based on seven domains of deprivation: income, employment, education, health, living environment, access to services and crime.^37^ LSOAs were grouped into national quintiles (quintile 1 is most deprived).
Morbidity: underlying cause of death and count of primary care medications	The underlying cause of death from the death certificate was used as a proxy for primary diagnosis, which is known to be an important factor associated with emergency hospitalisations towards the end of life.^10 38^ International classification of diseases, 10th revision codes were grouped into nine chronic illness causes of death, plus a category for ‘sudden causes’.^20 21^A count of unique primary care prescriptions dispensed in the 12–4 months before death was also used as a proxy for comorbidities, following an approach used elsewhere.^39^,^42^ We counted unique occurrences of British National Formulary seven-digit ‘subparagraph’ codes. Each code represents drugs in the same class, for example, code 0407020 indicates opioid analgesics; repeat prescriptions for the same or similar drugs were counted once only. The advantage of this method over alternative approaches, such as using hospital data to derive a comorbidity index based on diagnoses, is that it is not limited to people with hospital admissions and can be derived consistently for a whole cohort over a specified period (ie, predating our outcome of emergency visits in the last 3 months of life).

LAlocal authorityLSOAlower layer super output area

### Analysis

Counts and crude rates of emergency department visits per person in the last 3 months of life were used to describe the sample. To describe the intersection between ethnicity and area-based deprivation, we used regression models adjusted by age only, with an interaction between ethnicity and deprivation. From these models, we present the marginal, predicted mean rate per person of emergency department visits in the last 3 months of life (in-hours and out-of-hours combined), by ethnicity and deprivation, separately for men and women and colour-coded in a heatmap format.

Following an approach used elsewhere,^17^ we estimated the effect of ethnicity using four models, each controlling for additional covariates, to see how far the effects of ethnicity were explained by other variables. Models were run separately for men and women and separately for emergency department visits in-hours and out-of-hours. Model 1 is adjusted only for ethnicity and age; model 2 is additionally adjusted for population density and clustering at the local authority level; model 3 is additionally adjusted for area-based deprivation (with quintiles treated categorically to allow for non-linearity); model 4 is additionally adjusted for underlying causes of death and comorbidities based on a count of primary care medications (see table 1 for a summary of these covariates).

All models used complete cases and negative binomial regression to account for overdispersion in the outcome variable. We applied robust SEs to account for heteroskedasticity. For models that included geographical variables, SEs allowed for intragroup correlation (clustering) at the local authority level.^18^ Bar charts display the incidence rate ratio (IRR) for each model.

To evaluate the potential for unmeasured confounders that could explain away the main ethnicity effects, we report e-values for the final ‘model 4’. E-values are defined as the minimum strength of association on the risk ratio scale that an unmeasured confounder would need to have with both the exposure and the outcome to fully explain away an effect, in our case, the main effect of ethnicity on the outcome, conditional on the covariates.^19^

To investigate the effect of the COVID-19 pandemic on the association between ethnicity and the number of emergency department visits in the last 3 months of life, we modelled the interaction between year and ethnicity, adjusting only for age and sex to observe the effect of ethnicity without adjusting for other factors. This analysis was restricted to deaths between 1 July and 31 December 2020 and the same period in 2019. This time period ensured that for the 2020 deaths, emergency department visits occurred after the start of the first COVID-19 UK lockdown in March 2020.

### Sensitivity analysis

In a sensitivity analysis, we excluded people who died from sudden causes of death to limit the analysis to visits that were more likely to be avoidable. Non-sudden causes were defined following an established approach.^20 21^ In a second sensitivity analysis, we excluded people who died in a care home. Being a care home resident may reduce end-of-life emergency department visits and is less likely for some ethnic minority groups, which could explain some of the effect of ethnicity on emergency department visits.^12 22 23^

All analysis was carried out in Stata MP V.17; the analytical code is available here: https://github.com/BHFDSC/CCU024_02.

### Patient and public involvement

This study is part of the Marie Curie-funded Better End of Life Programme (MCSON-20–102) which has involved patients and the public throughout. Our patient and public involvement group suggested the initial idea for this study and were involved in the interpretation of the data.^4^

## Results

In England in 2020, 567 470 adult deaths were recorded; we excluded 540 decedents who were in hospital for the full duration of their last 3 months of life. The majority, 356 700 (62.9%), had at least one emergency department visit in the last 3 months of life. The crude rate of emergency department visits in the last 3 months of life was 1 per person. Descriptive data are described in table 2, and online supplemental table 1 gives a description of variables by ethnic group.

**Table 2:** Number of deaths, ED visits and crude rate of ED visits per person, in-hours and out-of-hours (evenings, weekends and bank holidays) in the last 3 months of life for deaths in England in 2020

	n of deaths*	Column %	n of ED visits	n of out-of-hours ED visits	n of in-hours ED visits	Overall rate of ED visits	Out-of-hours rate of ED visits	In-hours rate of ED visits
Total	566 930	100%	567 010	327 750	239 260	1.00	0.58	0.42
Median age(IQR)	82(72–89)							
Missing	6755	1.19%	2245	1380	865	0.33	0.20	0.13
Sex								
Men	285 185	50.3%	302 565	175 130	127 440	1.06	0.61	0.45
Women	279 140	49.2%	264 440	152 620	111 825	0.95	0.55	0.40
Other	10	0.0%	†	†	†	†	†	†
Missing	2595	0.5%	†	†	†	†	†	†
Ethnicity								
White British	494 260	87.2%	494 020	285 430	208 590	1.00	0.58	0.42
Black African	3065	0.5%	3430	1940	1495	1.12	0.63	0.49
Black Caribbean	5085	0.9%	5665	3240	2430	1.11	0.64	0.48
Bangladeshi	1680	0.3%	2155	1325	835	1.28	0.79	0.49
Pakistani	5130	0.9%	6235	3895	2345	1.22	0.76	0.46
Indian	8580	1.5%	10 025	5785	4240	1.17	0.67	0.49
Mixed	3320	0.6%	3505	2065	1445	1.06	0.62	0.43
Chinese	1040	0.2%	1035	610	425	0.99	0.59	0.41
White other	24 510	4.3%	25 025	14 245	10 780	1.02	0.58	0.44
Other	9765	1.7%	11 000	6420	4580	1.13	0.66	0.47
Missing	10 500	1.9%	4930	2815	2120	0.47	0.27	0.20
Underlying cause of death								
Malignant cancer	137 675	24.3%	141 540	77 695	63 850	1.03	0.56	0.46
Heart disease	83 870	14.8%	70 935	41 100	29 840	0.85	0.49	0.36
Respiratory disease	25 825	4.6%	32 615	18 720	13 900	1.26	0.72	0.54
Renal disease	4355	0.8%	4260	2410	1855	0.98	0.55	0.43
Liver disease	9000	1.6%	11 845	6715	5130	1.32	0.75	0.57
Dementia/Alzheimer’s/senility	73 695	13.0%	49 095	30 205	18 895	0.67	0.41	0.26
Neurodegenerative diseases	10 800	1.9%	9355	5655	3700	0.87	0.52	0.34
Stroke	27 590	4.9%	29 315	17 005	12 310	1.06	0.62	0.45
HIV	125	0.0%	145	90	60	1.19	0.72	0.47
Sudden causes	193 670	34.2%	217 575	127 975	89 600	1.12	0.66	0.46
Missing	345	0.1%	345	205	145	1.00	0.59	0.41
Count of medicines‡								
0–4	144 910	25.6%	127 500	71 915	55 590	0.88	0.50	0.38
5–8	154 580	27.3%	158 105	90 970	67 135	1.02	0.59	0.43
9–12	138 480	24.4%	143 035	83 370	59 665	1.03	0.60	0.43
13–54	128 965	22.7%	138 375	81 505	56 875	1.07	0.63	0.44
Area-based deprivation								
1 (most deprived)	119 485	21.1%	132 195	76 490	55 710	1.11	0.64	0.47
2	113 355	20.0%	119 835	69 275	50 565	1.06	0.61	0.45
3	115 115	20.3%	112 670	65 465	47 205	0.98	0.57	0.41
4	112 110	19.8%	107 265	61 890	45 375	0.96	0.55	0.40
5	103 145	18.2%	94 950	54 580	40 375	0.92	0.53	0.39
Missing	3725	0.7%	100	60	40	0.03	0.02	0.01
Population density‡								
2–1054	140 850	24.8%	129 775	75 700	54 075	0.92	0.54	0.38
1055–3138	140 795	24.8%	141 105	81 665	59 440	1.00	0.58	0.42
3139–5240	140 815	24.8%	144 220	83 525	60 700	1.02	0.59	0.43
5241– 106 716	140 745	24.8%	151 810	86 805	65 010	1.08	0.62	0.46
Missing	3725	0.7%	100	60	40	0.03	0.02	0.01

* Cell counts rounded up to nearest 5five to meet disclosure requirements from the data holding body.

† Supressed due to small cell counts of <10.

‡ Count of medicines and population density were continuous variables in the models but are summarised here using quartiles.

EDemergency department

**Figure 1:**
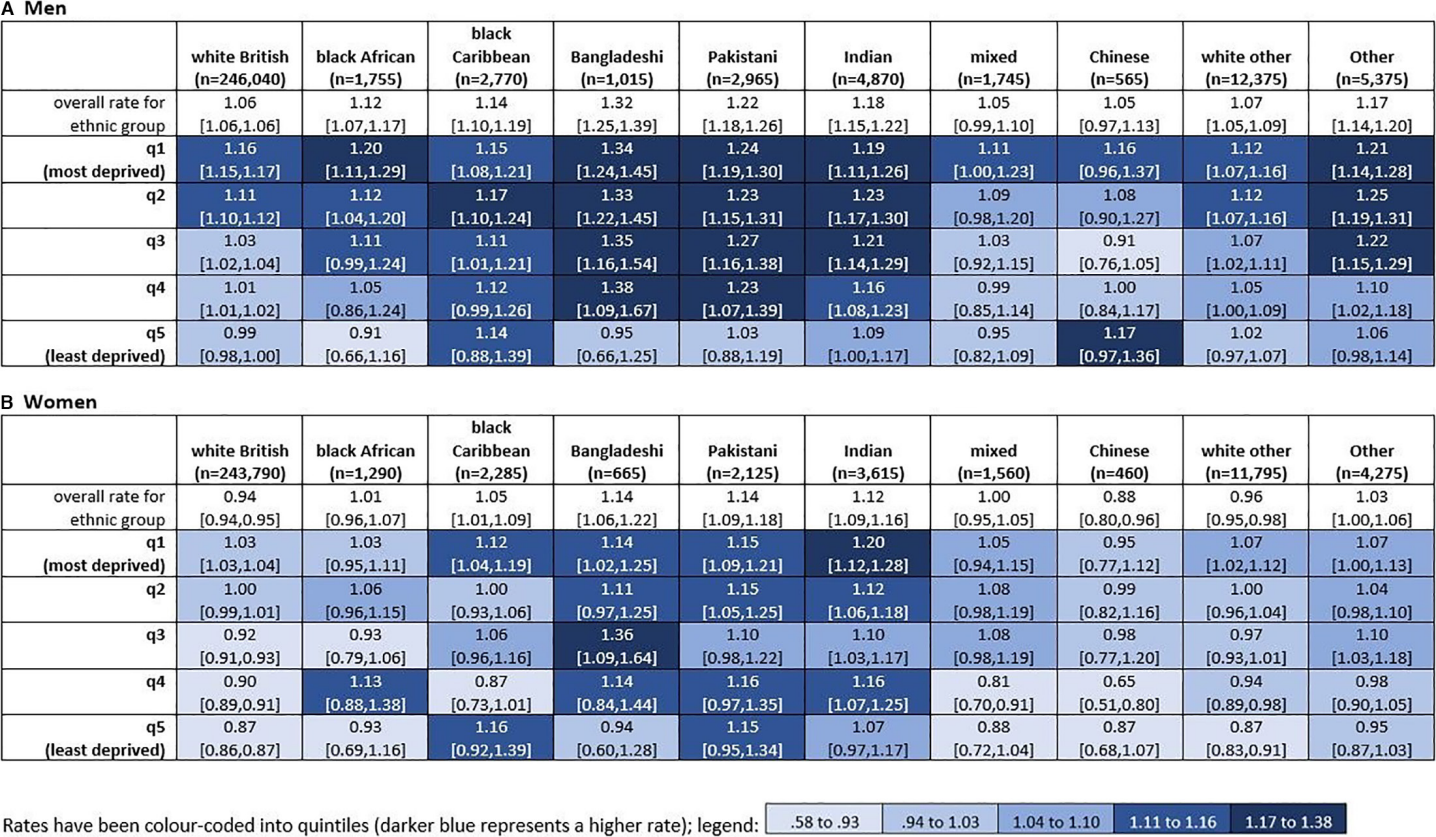
Age-adjusted predicted rate* per person of emergency department visits (in hours and out-of-hours combined) in the last 3 months of life, deaths in England in 2020, by ethnicity and level of area-based deprivation and gender. *Predicted mean rates are the marginal results from a negative binomial model adjusted for age with an interaction between ethnicity and deprivation.

A heat map of the marginal effects and the age-adjusted mean rate of emergency department visits in the last 3 months of life, by ethnicity and deprivation, separately for men and women are shown in figure 1. Bangladeshi and Pakistani men in quintiles 1–4 had the highest rates of emergency department visits in the last 3 months of life. For most groups (apart from Chinese men, black Caribbean women and Pakistani women), people in the most deprived quintile had a higher rate of emergency department visits than people in the least deprived quintile. The stepwise deprivation gradient seen for white British men and women, and black African men (ie, for each increase in area deprivation, there is an increase in the rate of emergency department visits), is not observed for most other ethnic groups.

### Model results

**Figure 2:**
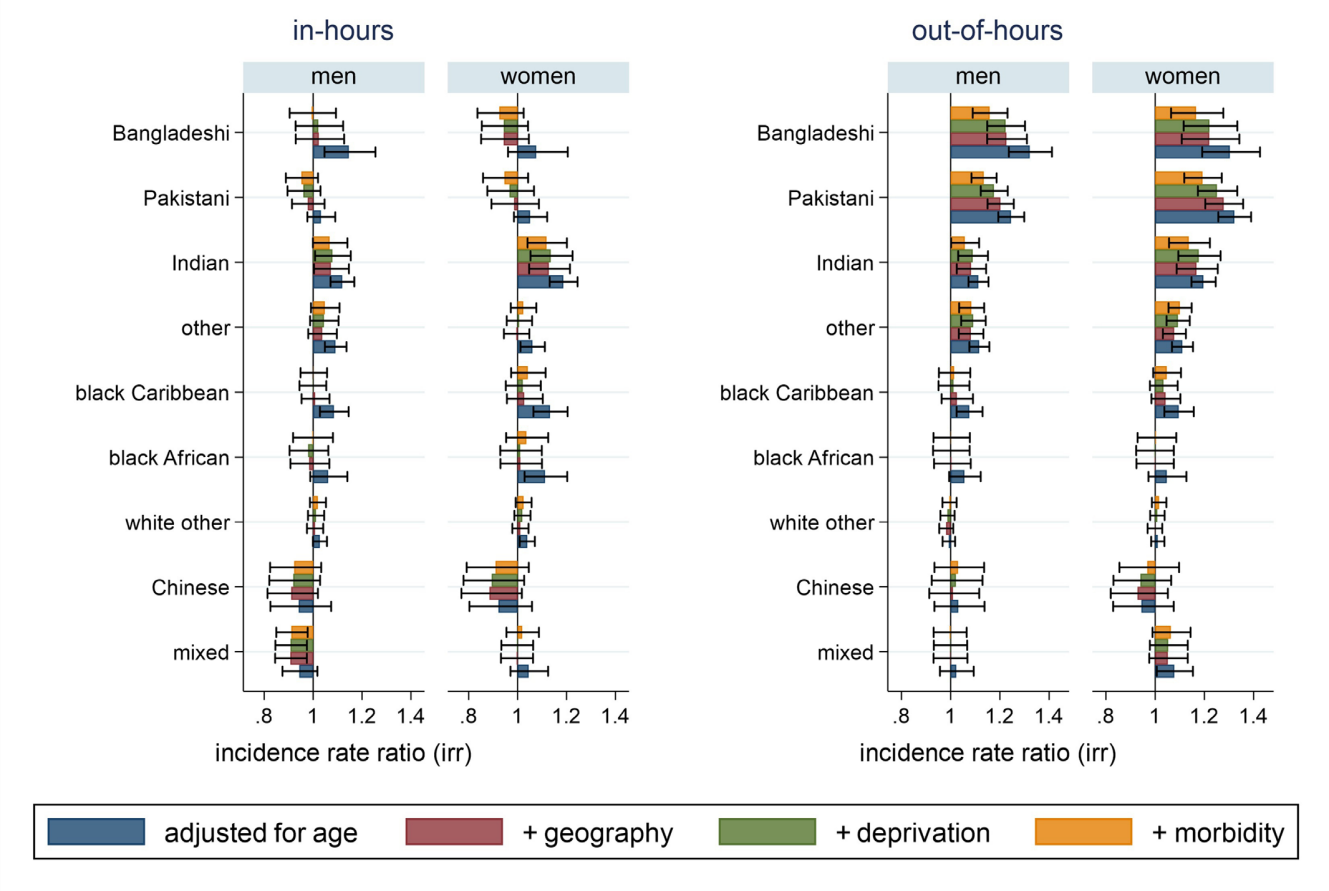
Adjusted incidence rate ratio and 95% CI of emergency department visits in last 3 months of life (in and out-of-hours), by ethnic group and sex, compared with white British, for all deaths in England in 2020. Notes: (1) ‘out-of-hours’ is defined as the period after 18:00 and before 08:00, weekends and bank holidays. (2) Negative binomial models adjusting for: model 1: age; model 2: geography (population density and robust SEs for clustering within local authorities); model 3: area-based deprivation (Index of Multiple Deprivation (2019) quintiles) and model 4: morbidity (underlying cause of death and count of unique primary care medications 12 to 4 months before death). (3) ‘Other’ ethnic groups include Asian other, Black other, Arab and other ethnic group categories. (4) For men: model 1, n=280 120; models 2 and 3, n=279 450; model 4, n=279 240. For women: model 1, n=272 175; models 2 and 3, n=271 840; model 4, n=271 725.

For in-hours emergency department visits, after adjusting for age (model 1), some ethnic groups, including Bangladeshi men, Indian men and women and black Caribbean men and women, had a statistically significant higher rate of visits compared with white British people. After adjusting for geography, area-based deprivation and morbidity (model 4), a statistically significant higher rate of emergency department visits is observed only for Indian women (figure 2 and online supplemental table 2).

For out-of-hours emergency department visits, after adjusting for age, geography, area-based deprivation and morbidity (model 4), men and women from Bangladeshi, Pakistani, Indian and ‘other’ ethnic groups have a statistically significant higher rate of visits compared with white British people (figure 2 and online supplemental table 3 for full model results).

For model 4, for out-of-hours emergency department visits, after adjusting for all the measured covariates, the e-values for the IRRs for Bangladeshi, Pakistani, Indian and ‘other’ men and women were 1.59, 1.53, 1.30, 1.39 and 1.61, 1.67, 1.53, 1.43, respectively. The relatively low e-values suggest a high likelihood that unmeasured confounders or mediators exist that could explain away the effects of ethnicity.

### Sensitivity analysis

In the sensitivity analysis, estimates for out-of-hours emergency department visits from model 4 (adjusted for age, geography, deprivation, and morbidity) tended to be strengthened in the sample excluding deaths from sudden causes and attenuated in the sample that also excluded deaths in care homes (online supplemental table 4).

### Interaction between year and ethnicity, comparing effects before and during the COVID-19 pandemic

This analysis included 260 370 people who died between July and December 2020 and 247 620 people who died in the same period in 2019 (see online supplemental table 5) for a descriptive summary of the age, sex, and ethnicity of these decedents. The age- and sex-adjusted rate of out-of-hours emergency department visits was lower for almost all ethnic groups (apart from for people with ‘mixed’ ethnicity) in 2020 than in 2019 (table 3). Compared with white British people, the gap in the out-of-hours emergency department visits rate was statistically significantly smaller in 2020 compared with 2019 for people with black African, Bangladeshi, Pakistani and Indian ethnicities than in 2019.

**Table 3:** Interaction effects for year and ethnicity on number of out-of-hours emergency department visits in the last 3 months of life for deaths between 1 July and 31 December in 2019 and 2020 and and the predicted (marginal) adjusted* mean rate of visits per death, by ethnicity and year

	Predicted rate in 2019(July–December)	Predicted rate in 2020(July–December)	‘Simple effects’(2020 minus 2019)	IRR interaction effects†(2019 is ref)
White British	0.61 (0.61–0.61)	0.59 (0.58–0.59)	−0.02 (–0.03 to –0.02)	Ref
Black African	0.72 (0.65–0.78)	0.59 (0.54–0.63)	−0.13 (–0.21 to –0.05)	0.85 (0.76–0.96)
Black Caribbean	0.70 (0.64–0.76)	0.62 (0.59–0.66)	−0.08 (–0.15 to –0.01)	0.92 (0.82–1.02)
Bangladeshi	0.96 (0.86–1.05)	0.75 (0.69–0.80)	−0.21 (–0.32 to –0.10)	0.81 (0.71–0.91)
Pakistani	0.80 (0.75–0.85)	0.70 (0.66–0.73)	−0.10 (–0.16 to –0.05)	0.90 (0.83–0.97)
Indian	0.75 (0.71–0.79)	0.65 (0.62–0.68)	−0.10 (–0.14 to –0.05)	0.90 (0.84–0.97)
Mixed	0.62 (0.57–0.67)	0.65 (0.60–0.70)	0.03 (–0.05 to 0.10)	1.08 (0.96–1.21)
Chinese	0.67 (0.58–0.75)	0.61 (0.54–0.68)	−0.06 (−0.17 to 0.06)	0.95 (0.79–1.13)
White other	0.63 (0.61–0.65)	0.59 (0.57–0.60)	−0.04 (−0.07 to –0.02)	0.97 (0.93–1.01)
Other	0.70 (0.67–0.72)	0.65 (0.62–0.68)	−0.05 (−0.08 to –0.01)	0.85 (0.76–0.96)

* Adjusted by age and sex only.

† Interaction effects are interpreted as the effect (IRR) for each ethnic group compared to white British in 2020 compared to 2019.

IRRincidence rate ratio

## Discussion

This study of all deaths in England in 2020 found that, compared with white British people, most ethnic minority groups have more emergency department visits in the last 3 months of life. The relationship between deprivation and emergency department visits varies by ethnicity, and ethnicity-related differences were most apparent for emergency department visits out-of-hours (evenings, weekends and bank holidays). For some ethnic groups, including people with black Caribbean and black African ethnicity, the higher number of emergency department visits out-of-hours was explained by differences in age, geography, area-based deprivation, and morbidity. However, for people with Bangladeshi, Pakistani, and Indian ethnicities, out-of-hours emergency department visits in the last 3 months of life remained higher for women and men compared with white British people after adjusting for age, geography, area-based deprivation, and morbidity.

Non-White ethnicity and living in a more deprived area have previously been identified as factors associated with more emergency department visits for people approaching the end of life with cancer, dementia, and in older adults.^10^,^12^ This study is the first to provide a more detailed breakdown of categories of ethnicity, to describe the intersection with deprivation and to highlight the out-of-hours period as important for understanding ethnicity-related differences in the use of emergency departments in the last months of life. People from ethnic minorities are more likely to live in densely populated areas with higher levels of area-based deprivation and to have more comorbidities.^15^ We found these factors were disproportionately prevalent among ethnic minority groups in our sample, and for some groups, they accounted for the higher number of emergency department visits. However, for people with Bangladeshi, Pakistani, and Indian ethnicities, these additional factors only partially explained the higher rate of out-of-hours visits in the last months of life, highlighting a need to understand the preferences, needs and experience of care for people with South Asian ethnicity living with terminal illness.

This study applied an intersectional lens to understand the social determinants of health service use.^15 17 24^ We found that living in the most deprived areas was associated with higher rates of emergency department visits for most ethnic groups. We identified a clear deprivation gradient for white British people, but this was not seen for most other ethnic minority groups. In part, this may be due to smaller populations but nevertheless reflects a less linear relationship between deprivation and emergency department visits for most ethnic minority groups. The results from our four models, each adjusting for additional explanatory factors, highlight the importance of understanding both the accumulation of social and clinical risk factors and recognising ethnicity as an independent explanatory factor.

Our sample of decedents has a lower proportion of people from ethnic minority groups than are alive in the general population because older populations in England tend to be less ethnically diverse. However, the population of people from ethnic minority groups in England is increasing, and like the majority White population, these populations are ageing.^25^ For example, of the 4 million people with Indian, Pakistani and Bangladeshi ethnicities living in England and Wales in 2021, 11% (450,000) were over 60 years old, an increase from 5% in 2011.^25 26^ Very little research has been done to understand the needs, cultural preferences and experiences of ethnic minority patients and families living with advanced illness.^27^ Some evidence suggests that Indian, Pakistani, and Bangladeshi communities may be less likely to access specialist palliative care,^28^ and when services are accessed, they may not meet the cultural and familial needs of patients from ethnic minorities.^29^ Previous work has identified considerable geographical variation in the provision of out-of-hours community-based care for patients with advanced illness, but this has not been explored in relation to ethnicity.^4^ The availability of out-of-hours telephone support lines, access to medicines out-of-hours and the cultural competency of these services are likely to be important factors in providing equitable community support.^4 30^ The findings from this study suggest that understanding the perspectives of minoritised groups and their needs and experiences, particularly in relation to out-of-hours care, should be a priority for future work.

Like other studies, we found a reduction in the number of emergency department visits during 2020 compared with 2019, reflecting pressure during the COVID-19 pandemic to avoid hospitalisations.^31^ Overall, patterns of differences in the number of emergency department visits between ethnic groups were similar in 2019 and 2020. However, some ethnic groups experienced larger reductions in the number of emergency department visits during the pandemic. People with black African, Bangladeshi, Pakistani and Indian ethnicities had the highest rates of emergency department visits in 2019 and saw the largest reductions during 2020. This may reflect a regression to the mean or could be due to patterns of behaviour such as greater resistance to, or fear of, hospitalisations among these ethnic groups during the COVID-19 pandemic.^32^ Monitoring post-COVID-19 trends and working to understand the causes of ethnicity-related differences in emergency department visits in the last months of life will be important for delivering more equitable care in the postpandemic recovery period.

Emergency department visits in the last months of life can be distressing and many may be avoidable, but some visits are needed and may be aligned with the preferences of patients and families.^5^ Our sensitivity analysis excluded deaths from sudden causes to concentrate on emergency department visits from patients with chronic illnesses, some of which would have been avoidable. In this sensitivity analysis, differences between ethnic groups were strengthened, suggesting that ethnicity-related differences in emergency department visit rates are driven more by chronic conditions than by visits associated with deaths from sudden causes. Previous models have conceptualised emergency department overcrowding as related to input, throughput, and output.^33^ Our study examines input only; further research that examines issues related to throughout and output, including investigations and treatments received in the emergency department and discharge from the emergency department, could help to understand more about the potential to improve care in the last months of life.

In a second sensitivity analysis, we excluded deaths in care homes, which attenuated but did not remove differences between ethnic groups, suggesting that lower rates of care home residence among some ethnic minority groups may partially explain their higher rate of emergency department visits.^22^ Care homes were disproportionately affected by COVID-19 infection, and the potential moderating effect of care home residence on ethnic inequality in emergency department visits warrants investigation in more recent data.

### Strengths and limitations

This whole-population study uses data on all deaths in England linked to hospital records, primary care prescription data, and primary care records.^16^ The linked data combines information on ethnicity from hospital records and primary care records to make use of a highly complete source of information on ethnicity.^16^ Our analysis shows previously undescribed differences between ethnic groups in the number of emergency department visits in the last 3 months of life. A limitation of the data is that ethnicity is likely to have been assigned rather than self-identified in many cases. We took a robust approach to measuring comorbidity through a count of unique primary care medications, generating a time-specific measure applicable to the full sample.

Of note, COVID-19 diagnosis was not included as a variable in this study because of low levels of testing and detection in the community during the first year of the pandemic. Although we adjusted for underlying causes of death and comorbidity, this does not include contributing causes of death or symptom burden. Variations in symptoms and associated levels of distress and anxiety are potentially important unmeasured confounders not accounted for in this analysis. We were not able to identify care home residents in our data, so to gain insight into the potential effect of care home residence, our sensitivity analysis excluded people who died in a care home. In England, in 2020, 80% of permanent care home residents who died, died in a care home.^34^ Therefore, death in a care home is a reasonable, although incomplete, proxy for care home resident status. Methods to identify care home residents from postcodes in routine data were not available to us but could be used in future studies to further explore care home residence as an explanatory factor.^35^

## Conclusions

This population-based study highlights that most ethnic minority groups have higher rates of emergency department visits in the last 3 months of life compared with white British people. These ethnicity-related differences emerge most strongly in the out-of-hours period (evenings, weekends and bank holidays), and patterns were similar during and before the COVID-19 pandemic. For people with Bangladeshi, Pakistani, and Indian ethnicities, their significantly higher rates of emergency department visits in the last 3 months of life were not explained by variation in geography, area-based deprivation, or morbidity. The populations of ethnic minorities in the UK are growing and ageing. We should make efforts to understand the needs, experiences and preferences of people from ethnic minorities living with terminal illness, in particular people from Bangladeshi, Pakistani, and Indian communities, to understand why these groups have higher rates of emergency department visits in the last months of life and whether this represents an inequity in access to community care.

## supplementary material

10.1136/bmjph-2024-001121 online supplemental file 1

10.1136/bmjph-2024-001121 online supplemental file 2

## Data Availability

Data may be obtained from a third party and are not publicly available.
