# Supplementary material for: Association between ethnicity and emergency department visits in the last three months of life in England: a retrospective population-based study using electronic health records
Source: BMJ Public Health. 2024 Oct 18;2(2):e001121. doi: 10.1136/bmjph-2024-001121 (PMC11816196; doi:10.1136/bmjph-2024-001121)
Supplement: online supplemental file 1 [file bmjph-2-2-s001.pdf]

Supplementary file for manuscript: Davies et al, Association between ethnicity and emergency department visits in the last three months of life in England: a retrospective population-based study using electronic health records

Table 1: Sample characteristics by ethnicity

	white British	black African	black Caribbean	Bangladeshi	Pakistani	Indian	mixed	Chinese	white other	other	missing
n	494680	3080	5095	1685	5135	8595	3335	1045	24555	9790	10505
**median age [IQ range]**	82 [73,89]	66 [54,79]	81 [68,87]	77 [62,84]	76 [63,84]	79 [67,86]	77 [60,86]	80 [67,87]	81 [69,88]	76 [62,86]	77 [66,86]
**sex**											
other	**	**	**	**	**	**	**	**	**	**	**
men	247145 (50.0%)	1765 (57.6%)	2790 (54.8%)	1015 (60.2%)	2980 (58.1%)	4910 (57.2%)	1755 (52.8%)	575 (55.0%)	12540 (51.2%)	5435 (55.7%)	4295 (40.9%)
women	247115 (50.0%)	1300 (42.4%)	2300 (45.2%)	670 (39.8%)	2150 (41.9%)	3675 (42.8%)	1570 (47.2%)	470 (45.0%)	11970 (48.8%)	4330 (44.3%)	3610 (34.4%)
missing	0	0	0	0	0	0	0	0	0	0	2595 (0.0%)
**underlying cause of death**											
malignant cancer	121625 (24.6%)	710 (23.2%)	1145 (22.5%)	305 (18.1%)	810 (15.8%)	1415 (16.5%)	830 (25.0%)	290 (27.8%)	6250 (25.5%)	2160 (22.1%)	2160 (20.6%)
heart disease	71740 (14.5%)	405 (13.2%)	735 (14.4%)	235 (13.9%)	890 (17.3%)	1620 (18.9%)	430 (12.9%)	130 (12.4%)	3765 (15.4%)	1445 (14.8%)	2495 (23.8%)
respiratory disease	23595 (4.8%)	45 (1.5%)	110 (2.2%)	65 (3.9%)	130 (2.5%)	175 (2.0%)	70 (2.1%)	25 (2.4%)	1015 (4.1%)	260 (2.7%)	355 (3.4%)
renal disease	3780 (0.8%)	40 (1.3%)	60 (1.2%)	20 (1.2%)	45 (0.9%)	95 (1.1%)	30 (0.9%)	10 (1.0%)	160 (0.7%)	85 (0.9%)	50 (0.5%)
liver disease	7700 (1.6%)	50 (1.6%)	50 (1.0%)	15 (0.9%)	60 (1.2%)	205 (2.4%)	55 (1.7%)	10 (1.0%)	485 (2.0%)	180 (1.8%)	210 (2.0%)
dementia/Alzheimers/ senility	66760 (13.5%)	170 (5.5%)	615 (12.1%)	100 (5.9%)	330 (6.4%)	670 (7.8%)	360 (10.8%)	95 (9.1%)	2960 (12.1%)	915 (9.4%)	735 (7.0%)
neurodegenerative diseases	9775 (2.0%)	30 (1.0%)	75 (1.5%)	20 (1.2%)	80 (1.6%)	135 (1.6%)	50 (1.5%)	15 (1.4%)	400 (1.6%)	140 (1.4%)	105 (1.0%)
stroke	23890 (4.8%)	175 (5.7%)	260 (5.1%)	115 (6.8%)	265 (5.2%)	440 (5.1%)	180 (5.4%)	75 (7.2%)	1185 (4.8%)	505 (5.2%)	515 (4.9%)
HIV	55 (0.0%)	30 (1.0%)	**	**	**	**	**	**	15 (0.1%)	15 (0.2%)	**
sudden causes	165080 (33.4%)	1420 (46.3%)	2050 (40.3%)	815 (48.4%)	2535 (49.4%)	3835 (44.7%)	1325 (39.8%)	410 (39.2%)	8275 (33.8%)	4070 (41.7%)	3870 (36.9%)
missing	280 (0.1%)	10 (0.3%)	**	**	**	**	**	**	15 (0.1%)	10 (0.1%)	20 (0.2%)

Table 1 continued: sample characteristics by ethnicity

	white British	black African	black Caribbean	Bangladeshi	Pakistani	Indian	mixed	Chinese	white other	other	missing
n	494680	3080	5095	1685	5135	8595	3335	1045	24555	9790	10505
**count of medicines**											
0 to 4	118930 (24.1%)	1325 (43.2%)	1485 (29.2%)	320 (19.0%)	1090 (21.2%)	2060 (24.0%)	1055 (31.7%)	415 (39.7%)	7360 (30.0%)	3280 (33.6%)	7610 (72.5%)
5 to 8	136960 (27.7%)	735 (24.0%)	1490 (29.3%)	355 (21.1%)	1205 (23.5%)	2135 (24.9%)	850 (25.6%)	285 (27.3%)	6390 (26.1%)	2625 (26.9%)	1565 (14.9%)
9 to 12	123730 (25.0%)	565 (18.4%)	1135 (22.3%)	455 (27.0%)	1245 (24.3%)	2045 (23.8%)	785 (23.6%)	205 (19.6%)	5500 (22.4%)	1975 (20.2%)	865 (8.2%)
13 to 54	114650 (23.2%)	445 (14.5%)	980 (19.3%)	565 (33.5%)	1595 (31.1%)	2350 (27.4%)	640 (19.2%)	140 (13.4%)	5270 (21.5%)	1890 (19.4%)	465 (4.4%)
**area-based deprivation**											
1 (most deprived)	100895 (20.4%)	1210 (39.5%)	2060 (40.5%)	780 (46.3%)	2730 (53.2%)	1820 (21.2%)	960 (28.9%)	225 (21.5%)	5145 (21.0%)	2300 (23.6%)	1380 (13.1%)
2	95935 (19.4%)	1035 (33.8%)	1660 (32.6%)	545 (32.3%)	1210 (23.6%)	2430 (28.3%)	830 (25.0%)	250 (23.9%)	5350 (21.8%)	2670 (27.3%)	1460 (13.9%)
3	101720 (20.6%)	430 (14.0%)	830 (16.3%)	205 (12.2%)	595 (11.6%)	1965 (22.9%)	680 (20.5%)	200 (19.1%)	4970 (20.3%)	1890 (19.4%)	1645 (15.7%)
4	101390 (20.5%)	245 (8.0%)	340 (6.7%)	95 (5.6%)	350 (6.8%)	1330 (15.5%)	470 (14.1%)	185 (17.7%)	4540 (18.5%)	1540 (15.8%)	1650 (15.7%)
5	93700 (19.0%)	140 (4.6%)	190 (3.7%)	65 (3.9%)	240 (4.7%)	995 (11.6%)	375 (11.3%)	175 (16.7%)	4315 (17.6%)	1295 (13.3%)	1665 (15.9%)
missing	630 (0.1%)	15 (0.5%)	**	**	15 (0.3%)	50 (0.6%)	15 (0.5%)	15 (1.4%)	200 (0.8%)	75 (0.8%)	2720 (25.9%)
**population density**											
2 to 1054	132175 (26.7%)	145 (4.7%)	145 (2.8%)	45 (2.7%)	140 (2.7%)	460 (5.4%)	395 (11.9%)	95 (9.1%)	4230 (17.3%)	855 (8.8%)	2180 (20.8%)
1055 to 3138	129115 (26.1%)	270 (8.8%)	485 (9.5%)	120 (7.1%)	640 (12.5%)	1285 (15.0%)	560 (16.8%)	185 (17.7%)	4840 (19.7%)	1380 (14.1%)	1935 (18.4%)
3139 to 5240	126600 (25.6%)	430 (14.0%)	805 (15.8%)	215 (12.8%)	975 (19.0%)	1750 (20.4%)	680 (20.5%)	235 (22.5%)	5545 (22.6%)	1805 (18.5%)	1800 (17.1%)
5241 to 106716	105745 (21.4%)	2215 (72.3%)	3650 (71.7%)	1310 (77.7%)	3360 (65.5%)	5040 (58.7%)	1680 (50.5%)	525 (50.2%)	9705 (39.6%)	5660 (58.0%)	1880 (17.9%)
missing	630 (0.1%)	15 (0.5%)	**	**	15 (0.3%)	50 (0.6%)	15 (0.5%)	15 (1.4%)	200 (0.8%)	75 (0.8%)	2720 (25.9%)

Table 2: Adjusted incidence rate ratio and 95% confidence interval of emergency department visits in last 3 months of life (in-hours), by ethnic group and sex, compared to white British, for all deaths in England in 2020

Note: *This table reports the estimates shown in the 'in-hours' section of figure 2 in the main manuscript*

	model 1 adjusted for age	model 2 + geography	model 3 + deprivation	model 4 + morbidity
black African	1.11 [1.03,1.20]	1.01 [0.93,1.10]	1.01 [0.93,1.10]	1.04 [0.95,1.12]
black Caribbean	1.13 [1.06,1.20]	1.03 [0.96,1.10]	1.02 [0.95,1.09]	1.04 [0.97,1.11]
Bangladeshi	1.08 [0.96,1.21]	0.94 [0.85,1.05]	0.94 [0.85,1.04]	0.93 [0.84,1.02]
Pakistani	1.05 [0.99,1.12]	0.99 [0.89,1.09]	0.97 [0.88,1.07]	0.95 [0.86,1.04]
Indian	1.19 [1.13,1.24]	1.13 [1.05,1.21]	1.14 [1.05,1.22]	1.12 [1.04,1.20]
mixed	1.04 [0.97,1.12]	1.00 [0.93,1.06]	1.00 [0.93,1.06]	1.02 [0.95,1.09]
Chinese	0.92 [0.80,1.06]	0.89 [0.77,1.02]	0.89 [0.78,1.03]	0.91 [0.79,1.05]
white other	1.04 [1.01,1.07]	1.01 [0.98,1.04]	1.02 [0.99,1.05]	1.02 [0.99,1.06]
other	1.06 [1.01,1.11]	0.99 [0.94,1.05]	1.01 [0.96,1.06]	1.02 [0.97,1.08]
black African	1.06 [0.99,1.14]	0.98 [0.91,1.07]	0.98 [0.90,1.06]	1.00 [0.92,1.08]
black Caribbean	1.08 [1.03,1.15]	1.01 [0.95,1.07]	1.00 [0.94,1.05]	1.00 [0.95,1.06]
Bangladeshi	1.15 [1.05,1.25]	1.02 [0.93,1.13]	1.02 [0.93,1.12]	0.99 [0.90,1.09]
Pakistani	1.03 [0.98,1.09]	0.98 [0.91,1.05]	0.96 [0.90,1.03]	0.95 [0.89,1.02]
Indian	1.12 [1.07,1.17]	1.07 [1.00,1.15]	1.08 [1.01,1.15]	1.07 [1.00,1.14]
mixed	0.94 [0.87,1.02]	0.91 [0.84,0.97]	0.91 [0.85,0.97]	0.91 [0.85,0.98]
Chinese	0.94 [0.83,1.07]	0.91 [0.81,1.02]	0.92 [0.82,1.03]	0.92 [0.82,1.03]
white other	1.03 [1.00,1.06]	1.01 [0.97,1.04]	1.01 [0.98,1.05]	1.02 [0.99,1.05]
other	1.09 [1.05,1.14]	1.04 [0.98,1.10]	1.04 [0.99,1.10]	1.05 [0.99,1.11]

For men: model 1 n=280,120; model 2 & 3 n=279,450; model 4 n=279,240. For women: model 1 n=272,175; model 2 & 3 n=271,840; model 4 n=271,725

Table 3: Adjusted incidence rate ratio and 95% confidence interval of emergency department visits in last 3 months of life (out-of-hours), by ethnic group and sex, compared to white British, for all deaths in England in 2020

Note: *This table reports the estimates shown in the 'out-of-hours' section of figure 2 in the main manuscript*

	model 1 adjusted for age	model 2 + geography	model 3 + deprivation	model 4 + morbidity
black African	1.05 [0.97,1.13]	1.00 [0.92,1.08]	1.00 [0.92,1.08]	1.00 [0.93,1.09]
black Caribbean	1.10 [1.04,1.16]	1.04 [0.98,1.10]	1.03 [0.98,1.09]	1.05 [0.99,1.11]
Bangladeshi	1.30 [1.19,1.43]	1.22 [1.11,1.34]	1.22 [1.12,1.33]	1.17 [1.06,1.28]
Pakistani	1.32 [1.26,1.39]	1.28 [1.20,1.36]	1.25 [1.17,1.33]	1.19 [1.12,1.27]
Indian	1.20 [1.15,1.25]	1.17 [1.09,1.25]	1.18 [1.09,1.27]	1.14 [1.06,1.22]
mixed	1.08 [1.01,1.15]	1.05 [0.98,1.13]	1.05 [0.98,1.13]	1.06 [0.99,1.14]
Chinese	0.94 [0.83,1.08]	0.93 [0.82,1.05]	0.94 [0.83,1.07]	0.97 [0.85,1.10]
white other	1.01 [0.98,1.04]	1.00 [0.97,1.03]	1.01 [0.98,1.04]	1.02 [0.99,1.05]
other	1.11 [1.07,1.15]	1.08 [1.03,1.12]	1.09 [1.05,1.14]	1.10 [1.05,1.15]
black African	1.06 [0.99,1.12]	1.00 [0.93,1.08]	1.00 [0.93,1.08]	1.00 [0.93,1.08]
black Caribbean	1.08 [1.02,1.13]	1.03 [0.96,1.09]	1.01 [0.95,1.08]	1.01 [0.95,1.08]
Bangladeshi	1.32 [1.24,1.41]	1.23 [1.15,1.31]	1.22 [1.15,1.30]	1.16 [1.09,1.23]
Pakistani	1.25 [1.19,1.30]	1.20 [1.15,1.26]	1.18 [1.12,1.23]	1.13 [1.08,1.19]
Indian	1.11 [1.07,1.15]	1.08 [1.02,1.14]	1.09 [1.03,1.15]	1.06 [1.00,1.12]
mixed	1.02 [0.96,1.09]	1.00 [0.93,1.07]	1.00 [0.93,1.07]	1.00 [0.93,1.06]
Chinese	1.03 [0.93,1.14]	1.01 [0.91,1.12]	1.02 [0.92,1.13]	1.03 [0.93,1.14]
white other	0.99 [0.97,1.02]	0.98 [0.95,1.01]	0.99 [0.96,1.02]	0.99 [0.97,1.02]
other	1.12 [1.08,1.16]	1.08 [1.03,1.13]	1.09 [1.04,1.14]	1.08 [1.03,1.14]

For men: model 1 n=280,120; model 2 & 3 n=279,450; model 4 n=279,240. For women: model 1 n=272,175; model 2 & 3 n=271,840; model 4 n=271,725

Table 3.1: Model 4, full model results (for women, n= 271,725) for out-of-hours emergency department visits in the last 3 months of life adjusted by age, geography, area-based deprivation and morbidity, for all deaths in England in 2020

Note: this table reports the full model results for women the estimates shown in the out-of-hours section of figure 2 in the main manuscript

	IRR	Std error	Lower CI	Upper CI
**ethnicity**				
white British	ref			
black African	1.00	0.04	0.93	1.09
black Caribbean	1.05	0.03	0.99	1.11
Bangladeshi	1.17	0.05	1.06	1.28
Pakistani	1.19	0.04	1.12	1.27
Indian	1.14	0.04	1.06	1.22
mixed	1.06	0.04	0.99	1.14
Chinese	0.97	0.06	0.85	1.10
white other	1.02	0.02	0.99	1.05
other	1.10	0.02	1.05	1.15
missing	1.00	0.00	0.99	1.00
**people per sqkm**	1.00	0.00	1.00	1.00
**area-based deprivation**				
1 (most deprived)	ref			
2	0.98	0.01	0.95	1.00
3	0.92	0.01	0.89	0.95
4	0.90	0.01	0.87	0.93
5	0.86	0.02	0.83	0.90
**underlying cause of death**				
malignant cancer	ref			
heart disease	1.01	0.01	0.98	1.04
respiratory disease	1.32	0.02	1.28	1.36
renal disease	1.03	0.03	0.96	1.09
liver disease	1.27	0.03	1.21	1.34
dementia/Alzheimers/senility	0.72	0.01	0.70	0.74
neurodegenerative diseases	0.86	0.02	0.82	0.91
stroke	1.14	0.02	1.10	1.17
HIV	1.22	0.25	0.82	1.82
sudden causes	1.22	0.01	1.19	1.25
**count of distinct medications**	1.01	0.00	1.01	1.01
**_cons**	0.74	0.02	0.70	0.78

Table 3.2: Model 4, full model results (for men, n= 279,240) for out-of-hours emergency department visits in the last 3 months of life adjusted by age, geography, area-based deprivation and morbidity, for all deaths in England in 2020

Note: this table reports the full model results for men the estimates shown in the out-of-hours section of figure 2 in the main manuscript

**ethnicity**	IRR	Std error	Lower CI	Upper CI
white British	ref			
black African	1.00	0.04	0.93	1.08
black Caribbean	1.01	0.03	0.95	1.08
Bangladeshi	1.16	0.04	1.09	1.23
Pakistani	1.13	0.03	1.08	1.19
Indian	1.06	0.03	1.00	1.12
mixed	1.00	0.03	0.93	1.06
Chinese	1.03	0.05	0.93	1.14
white other	0.99	0.01	0.97	1.02
other	1.08	0.03	1.03	1.14
missing	1.00	0.00	1.00	1.00
**people per sqkm**	1.00	0.00	1.00	1.00
**area-based deprivation**				
1 (most deprived)	ref			
2	0.97	0.01	0.95	0.99
3	0.93	0.01	0.90	0.95
4	0.91	0.01	0.88	0.93
5	0.88	0.02	0.85	0.92
**underlying cause of death**				
malignant cancer	ref			
heart disease	0.80	0.01	0.78	0.82
respiratory disease	1.15	0.02	1.12	1.19
renal disease	0.95	0.03	0.90	1.01
liver disease	1.33	0.03	1.28	1.39
dementia/Alzheimers/senility	0.84	0.01	0.81	0.86
neurodegenerative diseases	0.92	0.02	0.89	0.96
stroke	1.10	0.02	1.07	1.13
HIV	1.23	0.15	0.97	1.56
sudden causes	1.14	0.01	1.12	1.16
**count of distinct medications**	1.02	0.00	1.01	1.02
**_cons**	0.48	0.01	0.45	0.50

Table 4: sensitivity analysis, main ethnicity effects (IRR compared to white British) for the sample excluding sudden causes of death and the sample additionally excluding deaths in care homes

		excluding deaths from sudden causes<br>women (n=177,815); men (n=171,980)	additionally excluding deaths in care homes<br>women (n=120,260); men (n=137,735)
**women**	black African	1.08 [0.97,1.20]	1.09 [0.98,1.21]
	black Caribbean	1.06 [0.99,1.14]	1.05 [0.97,1.13]
	Bangladeshi	1.35 [1.16,1.58]	1.25 [1.06,1.48]
	Pakistani	1.25 [1.15,1.36]	1.16 [1.06,1.27]
	Indian	1.17 [1.06,1.29]	1.11 [1.00,1.23]
	mixed	1.09 [0.99,1.21]	1.09 [0.99,1.21]
	Chinese	1.00 [0.84,1.20]	1.01 [0.84,1.22]
	white other	1.04 [1.00,1.07]	1.02 [0.98,1.06]
	other	1.12 [1.06,1.19]	1.10 [1.04,1.16]
**men**	black African	1.06 [0.93,1.20]	1.07 [0.94,1.22]
	black Caribbean	1.09 [1.01,1.19]	1.09 [1.00,1.18]
	Bangladeshi	1.21 [1.10,1.33]	1.14 [1.04,1.26]
	Pakistani	1.22 [1.14,1.29]	1.16 [1.09,1.23]
	Indian	1.12 [1.04,1.21]	1.08 [1.00,1.17]
	mixed	1.01 [0.92,1.11]	1.00 [0.91,1.10]
	Chinese	0.99 [0.85,1.15]	0.95 [0.81,1.11]
	white other	1.01 [0.98,1.05]	1.02 [0.98,1.06]
	other	1.15 [1.07,1.24]	1.16 [1.07,1.26]

Table 5.1: Number* of deaths, emergency department (ED) visits, and rates per person of emergency department visits in-hours and out-of-hours (evenings, weekends and bank holidays) in the last 3 months of life, for deaths in England between July and December 2019

	n of deaths	n of ED visits	n of out-of-hours ED visits	n of in-hours ED visits	overall rate of ED visits	out-of-hours rate of ED visits	in-hours rate of ED visits
median age [IQ range]	82 [71 to 89]						
**sex**							
other	10	**	**	**	**	**	**
men	122740	136140	78535	57605	1.11	0.64	0.47
women	123160	123630	71445	52185	1.00	0.58	0.42
missing	1715	**	**	**	**	**	**
**ethnicity**							
white British	216775	228045	131645	96400	1.05	0.61	0.44
black African	1015	1310	750	565	1.29	0.74	0.55
black Caribbean	1740	2195	1220	975	1.26	0.70	0.56
Bangladeshi	475	745	465	285	1.57	0.97	0.59
Pakistani	1650	2215	1345	875	1.34	0.81	0.53
Indian	3095	3980	2335	1650	1.29	0.75	0.53
mixed	1315	1385	820	565	1.05	0.63	0.43
Chinese	395	460	265	195	1.17	0.67	0.50
white other	9545	10555	6035	4525	1.11	0.63	0.47
other	4405	5390	3115	2280	1.22	0.71	0.52
missing	7235	3520	2015	1505	0.49	0.28	0.21

*cell counts rounded up to nearest 5 to meet disclosure requirements from the data holding body; **supressed due to small cell counts of <10;

Table 5.2: Number* of deaths, emergency department (ED) visits, and rates per person of emergency department visits in-hours and out-of-hours (evenings, weekends and bank holidays) in the last 3 months of life, for deaths in England between July and December 2020

	n of deaths	n of ED visits	n of out-of-hours ED visits	n of in-hours ED visits	overall rate of ED visits	out-of-hours rate of ED visits	in-hours rate of ED visits
median age [IQ range]	81 [72 to 89]						
**sex**							
other	**	**	**	**	**	**	**
men	131730	143140	81930	61210	1.09	0.62	0.46
women	127555	123850	70550	53300	0.97	0.55	0.42
missing	1085	**	**	**	**	**	**
**ethnicity**							
white british	227785	233635	133415	100225	1.03	0.59	0.44
black african	1290	1450	785	670	1.12	0.61	0.52
black caribbean	2225	2435	1390	1050	1.09	0.62	0.47
bangladeshi	835	1045	635	410	1.25	0.76	0.49
pakistani	2595	3000	1840	1160	1.16	0.71	0.45
indian	3670	4225	2430	1795	1.15	0.66	0.49
mixed	1240	1420	815	605	1.14	0.66	0.49
chinese	400	425	245	180	1.06	0.62	0.44
white other	12695	13335	7495	5840	1.05	0.59	0.46
other	3830	4390	2540	1855	1.15	0.66	0.48
missing	3820	1645	910	740	0.43	0.24	0.19

*cell counts rounded up to nearest 5 to meet disclosure requirements from the data holding body; **supressed due to small cell counts of <10;
