# Supplementary material for: Association between ethnicity and emergency department visits in the last three months of life in England: a retrospective population-based study using electronic health records
Source: BMJ Public Health. 2024 Oct 18;2(2):e001121. doi: 10.1136/bmjph-2024-001121 (PMC11816196; doi:10.1136/bmjph-2024-001121)
Supplement: online supplemental file 2 [file bmjph-2-2-s002.docx]

| Institution | Member Name |
| --- | --- |
| Addenbrooke's Hospital | Jon Boyle |
| Barts Health NHS Trust | Alastair Proudfoot |
| Barts Health NHS Trust | Andrew Constantine |
| Barts Health NHS Trust | Dan Jones |
| Barts Health NHS Trust | Krishnaraj Rathod |
| Barts Health NHS Trust | Nida Ahmed |
| Barts Health NHS Trust | Richard Fitzgerald |
| British Heart Foundation | Dan O’Connell |
| British Heart Foundation | Naomi Herz |
| British Heart Foundation | Rony Arafin |
| British Heart Foundation | Sonya Babu-Narayan |
| British Heart Foundation | Zainab Karim |
| Cancer Research UK | Jon Shelton |
| Cancer Research UK | Martina Slapkova |
| Cancer Research UK | Rosie Hinchliffe |
| Cancer Research UK | Shane Johnson |
| Cardiff Metropolitan University | Renin Toms |
| Cardiff University | Julia Townson |
| European Bioinformatics Institute | Ewan Birney |
| European Bioinformatics Institute | Moritz Gerstung |
| Great Ormond Street Hospital | Katherine Brown |
| Guy’s and St Thomas’ NHS Foundation Trust | Benjamin Zuckerman |
| Guy’s and St Thomas’ NHS Foundation Trust | Ernest Wong |
| Guy's and St Thomas' NHS Foundation Trust | Tasanee Braithwaite |
| Health Data Research UK/BHF Data Science Centre | Anna Stevenson |
| Health Data Research UK/BHF Data Science Centre | Annette Jackson |
| Health Data Research UK/BHF Data Science Centre | Cathie Sudlow |
| Health Data Research UK/BHF Data Science Centre | Fionna Chalmers |
| Health Data Research UK/BHF Data Science Centre | Jadene Lewis |
| Health Data Research UK/BHF Data Science Centre | James Farrell |
| Health Data Research UK/BHF Data Science Centre | Jemma Austin |
| Health Data Research UK/BHF Data Science Centre | John Nolan |
| Health Data Research UK/BHF Data Science Centre | Kate McAllister |
| Health Data Research UK/BHF Data Science Centre | Lars Murdock |
| Health Data Research UK/BHF Data Science Centre | Lynn Morrice |
| Health Data Research UK/BHF Data Science Centre | Mehrdad Mizani |
| Health Data Research UK/BHF Data Science Centre | Melissa Webb |
| Health Data Research UK/BHF Data Science Centre | Ross Forsyth |
| Health Data Research UK/BHF Data Science Centre | Rouven Priedon |
| Health Data Research UK/BHF Data Science Centre | Samaira Khan |
| Health Data Research UK/BHF Data Science Centre | Steffen Petersen |
| Health Data Research UK/BHF Data Science Centre | Thomas Bolton |
| Health Data Research UK/BHF Data Science Centre | Zach Welshman |
| Healthcare Quality Improvement Partnership | Caroline Rogers |
| Imperial College London | Alun Davies |
| Imperial College London | Arunashis Sau |
| Imperial College London | Costas Kallis |
| Imperial College London | Fu Siong Ng |
| Imperial College London | Hannah Whittaker |
| Imperial College London | Ioanna Tzoulaki |
| Imperial College London | Jennifer Quint |
| Imperial College London | Juliette Unwin |
| Imperial College London | Libor Pastika |
| Imperial College London | Petter Brodin |
| Imperial College London | Philip Stone |
| Imperial College London | Safa Salim |
| Imperial College London | Sarah Cook |
| Imperial College London | Sarah Onida |
| Imperial College London (LCP) | Alistair Marsland |
| Imperial College London (LCP) | Andrew Thompson |
| Imperial College London (LCP) | Sara Holloway |
| Imperial College London (LCP) | Thomas Porter |
| INSIGHT | Alastair Denniston |
| Keele University | Mamas Mamas |
| King's College London | Abdel Douiri |
| King's College London | Adejoke Oluyase |
| King's College London | Ajay Shah |
| King's College London | Alexandru Dregan |
| King’s College London | Amy Ronaldson |
| King's College London | Anna Bone |
| King's College London | Antonio Cannata |
| King's College London | Ben Bray |
| King's College London | Charles Wolfe |
| King's College London | Daniel Bromage |
| King's College London | Dominic Oliver |
| King's College London | Elena Nikiphorou |
| King's College London | Emeka Chukwusa |
| King's College London | Gareth Williams |
| King's College London | Gayan Perera |
| King's College London | Harry Watson |
| King's College London | Irene Higginson |
| King's College London | Javiera Leniz Martelli |
| King's College London | Jayati Das-Munshi |
| King's College London | Joanna Davies |
| King's College London | Johnny Downs |
| King's College London | Katherine Sleeman |
| King's College London | Mevhibe Hocaoglu |
| King’s College London | Natasha Chilman |
| King's College London | Rachel Cripps |
| King's College London | Richard Killick |
| King's College London | Theresa McDonagh |
| King's College London | Vasa Curcin |
| Leeds Teaching Hospitals NHS Trust | Carin van Doorn |
| London School of Economics & Political Science | Rocco Friebel |
| London School of Hygiene & Tropical Medicine | Arturo de la Cruz |
| London School of Hygiene & Tropical Medicine | Dorothea Nitsch |
| London School of Hygiene & Tropical Medicine | Patrick Bidulka |
| London School of Hygiene & Tropical Medicine | Qiuju Li |
| Manchester University NHS Foundation Trust | Martin Rutter |
| Newcastle-upon-Tyne Hospitals NHS Foundation Trust | Alex Grundmann |
| NHS England | Adam Hollings |
| NHS England | Angeliki Antonarou |
| NHS England | Daniel Schofield |
| NHS England | Deborah Lowe |
| NHS England | Elizabeth Kelly |
| NHS England | Richardson |
| NHS England | Humaira Hussein |
| NHS England | Jake Kasan |
| NHS England | Nickie Wareing |
| NHS England | Russell Healey |
| NHS England | Shoaib Ali Ajaib |
| NHS Lanarkshire | Mark Barber |
| NHS Scotland | Carole Morris |
| NICE | Felix Greaves |
| NICE | Jennifer Beveridge |
| NICE | Seamus Kent |
| NICE | Thomas Lawrence |
| NICE | Vandana Ayyar-Gupta |
| Office for National Statistics | Camille Harrison |
| Office for National Statistics | Myer Glickman |
| Office for National Statistics | Vahé Nafilyan |
| Queen Mary University of London | Deepti Gurdasani |
| Queen's University Belfast | Frank Kee |
| Royal Brompton and Harefield Hospitals | Paz Tayal |
| Royal College of Surgeons of England | David Cromwell |
| Royal Free London NHS Foundation Trust | Amar Shah |
| Royal Free London NHS Foundation Trust | Swapna Mandal |
| Royal Papworth Hospital NHS Foundation Trust | Florian Falter |
| Royal Papworth Hospital NHS Foundation Trust | Joseph Newman |
| Royal United Hospitals Bath NHS Foundation Trust | Jennifer Rossdale |
| St George’s University of London | Elijah Behr |
| St George’s University of London | Nuria Sanchez |
| St George’s University of London | Xinkai Wang |
| Swansea Bay University Health Board | Daniel Harris |
| Swansea University | Amanda Marchant |
| Swansea University | Ashley Akbari |
| Swansea University | Daniel King |
| Swansea University | David Powell |
| Swansea University | Elizabeth A Ellins |
| Swansea University | Fatemeh Torabi |
| Swansea University | Gareth Davies |
| Swansea University | Hoda Abbasizanjani |
| Swansea University | Huw Strafford |
| Swansea University | Jane Lyons |
| Swansea University | Julian Halcox |
| Swansea University | Laura North |
| Swansea University | Marcos del Pozo Banos |
| Swansea University | Owen Pickrell |
| Swansea University | Ronan Lyons |
| Swansea University - DATAMIND | Ann John |
| University College | Robert Aldridge |
| University College London | Abraham Olvera-Barrios |
| University College London | Adnan Tufail |
| University College London | Alasdair Warwick |
| University College London | Alex Handy |
| University College London | Alvina Lai |
| University College London | Ami Banerjee |
| University College London | Ana Torralbo |
| University College London | Ana-Catarina Pinho-Gomes |
| University College London | Andrej Ivanovic |
| University College London | Andrew Lambarth |
| University College London | Anthony Khawaja |
| University College London | Ashkan Dashtban |
| University College London | Becky White |
| University College London | Christina Pagel |
| University College London | Christopher Tomlinson |
| University College London | Chu Siyu |
| University College London | David Selby |
| University College London | Eloise Withnell |
| University College London | Emma Whitfield |
| University College London | Eva Keller |
| University College London | Evaleen Malgapo |
| University College London | Ferran Espuny-Pujol |
| University College London | Flavien Hardy |
| University College London | Floriaan Schmidt |
| University College London | Freya Allery |
| University College London | Harry Hemingway |
| University College London | Honghan Wu |
| University College London | Jinge Wu |
| University College London | Johan Thygesen |
| University College London | Johannes Heyl |
| University College London | Kate Cheema |
| University College London | Katie Harron |
| University College London | Ken Li |
| University College London | Kerrie Stevenson |
| University College London | Laura Pasea |
| University College London | Louise Choo |
| University College London | Luca Grieco |
| University College London | Manuel Gomes |
| University College London | Matt Sydes |
| University College London | Mehrdad Mizani |
| University College London | Michalis Katsoulis |
| University College London | Mohamed Mohamed |
| University College London | Naomi Launders |
| University College London | Nushrat Khan |
| University College London | Paula Lorgelly |
| University College London | Pedro Machado |
| University College London | Pia Hardelid |
| University College London | Qi Huang |
| University College London | Ravi Shankar |
| University College London | Riyaz Patel |
| University College London | Roy Schwartz |
| University College London | Rui Providencia |
| University College London | Ruth Gilbert |
| University College London | Sam Quill |
| University College London | Samuel Kim |
| University College London | Simon Ellershaw |
| University College London | Sonya Crowe |
| University College London | Spiros Denaxas |
| University College London | Tuankasfee Hama |
| University College London | Waty Lilaonitkul |
| University College London | Yat Yi Fan |
| University College London | Yi Mu |
| University College London | Yoryos Lyratzopoulos |
| University College London / DATAMIND | David Osborn |
| University Hospital Bristol NHS Foundation Trust | Serban Stoica |
| University Hospital of North Midlands | Arun Pherwani |
| University of Aberdeen | Mary Joan Macleod |
| University of Birmingham | Sarah Wang |
| University of Birmingham and Queen Elizabeth Hospital Birmingham | Mark Thomas |
| University of Bristol | Arun Karthikeyan Suseeladevi |
| University of Bristol | Ben Gibbison |
| University of Bristol | Dann Mitchell |
| University of Bristol | Deborah Lawler |
| University of Bristol | Eleanor Walsh |
| University of Bristol | Elsie Horne |
| University of Bristol | Ewan Walker |
| University of Bristol | Gianni Angelini |
| University of Bristol | Jeremy Chan |
| University of Bristol | John Macleod |
| University of Bristol | Jonathan Sterne |
| University of Bristol | Katharine Looker |
| University of Bristol | Kurt Taylor |
| University of Bristol | Livia Pierotti |
| University of Bristol | Luisa Zuccolo |
| University of Bristol | Martha Elwenspoek |
| University of Bristol | Marwa Al Arab |
| University of Bristol | Massimo Caputo |
| University of Bristol | Mira Hidajat |
| University of Bristol | Neil Davies |
| University of Bristol | Paul Madley-Dowd |
| University of Bristol | Rachel Denholm |
| University of Bristol | Rochelle Knight |
| University of Bristol | Rupert Payne |
| University of Bristol | Shubhra Sinha |
| University of Bristol | Teri-Louise North |
| University of Bristol | Tim Dong |
| University of Bristol | Tom Palmer |
| University of Bristol | Venexia Walker |
| University of Bristol | Yueying Li |
| University of Cambridge | Alexia Sampri |
| University of Cambridge | Angela Wood |
| University of Cambridge | Carmen Petitjean |
| University of Cambridge | Chimweta Chilala |
| University of Cambridge | Chriselda Oliver |
| University of Cambridge | David Brind |
| University of Cambridge | Elena Raffetti |
| University of Cambridge | Elias Allara |
| University of Cambridge | Emanuele Di Angelantonio |
| University of Cambridge | Eoin McKinney |
| University of Cambridge | Fabian Falck |
| University of Cambridge | Genevieve Cezard |
| University of Cambridge | Hannah Harrison |
| University of Cambridge | Haoting Zhang |
| University of Cambridge | Isabel Walter |
| University of Cambridge | Jessica Barrett |
| University of Cambridge | John Danesh |
| University of Cambridge | John Ford |
| University of Cambridge | Katie Saunders |
| University of Cambridge | Lisa Pennells |
| University of Cambridge | Lois Kim |
| University of Cambridge | Mike Inouye |
| University of Cambridge | Robert Fletcher |
| University of Cambridge | Rutendo Mapeta |
| University of Cambridge | Samantha Ip |
| University of Cambridge | Spencer Keene |
| University of Cambridge | Stelios Boulitsakis Logothetis |
| University of Cambridge | Stephen Kaptoge |
| University of Cambridge | Tianxiao Wang |
| University of Cambridge | Tom Pape |
| University of Cambridge | Wen Shi |
| University of Cambridge | Xilin Jiang |
| University of Cambridge | Xiyun Jiang |
| University of Cambridge | Yanfan Li |
| University of Dundee | Daniel Morales |
| University of Dundee | David Moreno Martos |
| University of Dundee | Huan Wang |
| University of Dundee | Ify Mordi |
| University of Dundee | Samira Bell |
| University of Edinburgh | Alan Carson |
| University of Edinburgh | Alice Hosking |
| University of Edinburgh | Annemarie Docherty |
| University of Edinburgh | Athina Spiliopoulou |
| University of Edinburgh | Baljean Dhillon |
| University of Edinburgh | Carlos Sanchez Soriano |
| University of Edinburgh | Caroline Jackson |
| University of Edinburgh | Christian Schnier |
| University of Edinburgh | Claire Tochel |
| University of Edinburgh | Gwenetta Curry |
| University of Edinburgh | Helen Colhoun |
| University of Edinburgh | Huayu Zhang |
| University of Edinburgh | Joe Mellor |
| University of Edinburgh | Laura Sherlock |
| University of Edinburgh | Luke Blackbourn |
| University of Edinburgh | Michelle Williams |
| University of Edinburgh | Miguel Bernabeu Llinares |
| University of Edinburgh | Niamh McLennan |
| University of Edinburgh | Rebecca Reynolds |
| University of Edinburgh | Richard Chin |
| University of Edinburgh | Steven Kerr |
| University of Edinburgh | Tim Wilkinson |
| University of Edinburgh | Verónica Cabreira |
| University of Edinburgh | William Berthon |
| University of Edinburgh | William Whiteley |
| University of Exeter | John Dennis |
| University of Exeter | Michael Allen |
| University of Glasgow | Angela Henderson |
| University of Glasgow | Clea du Toit |
| University of Glasgow | Colin Berry |
| University of Glasgow | Craig Melville |
| University of Glasgow | Deborah Kinnear |
| University of Glasgow | Dennis Tran |
| University of Glasgow | Filip Sosenko |
| University of Glasgow | Frederick Ho |
| University of Glasgow | Jill Pell |
| University of Glasgow | Jocelyn Friday |
| University of Glasgow | John Cleland |
| University of Glasgow | Naveed Sattar |
| University of Glasgow | Salil Deo |
| University of Glasgow | Sandosh Padmanabhan |
| University of Glasgow | Terry Quinn |
| University of Leeds | Jianhua Wu |
| University of Leeds | Ramesh Nadarajah |
| University of Leicester | Anna Hansell |
| University of Leicester | Anvesha Singh |
| University of Leicester | Cameron Razieh |
| University of Leicester | Claire Lawson |
| University of Leicester | Clare Gillies |
| University of Leicester | Francesco Zaccardi |
| University of Leicester | Iain Squire |
| University of Leicester | Kamlesh Khunti |
| University of Leicester | Matthew Bown |
| University of Leicester | Muhammad Rashid |
| University of Leicester | Sharmin Shabnam |
| University of Leicester | Shirley Sze |
| University of Leicester | Tom Yates |
| University of Leicester | Yogini Chudasama |
| University of Liverpool | Andrew Mason |
| University of Liverpool | Benedict Michael |
| University of Liverpool | Caroline Dale |
| University of Liverpool | David Hughes |
| University of Liverpool | Maria Sudell |
| University of Liverpool | Mark Green |
| University of Liverpool | Munir Pirmohamed |
| University of Liverpool | Pardis Biglarbeigi |
| University of Liverpool | Reecha Sofat |
| University of Liverpool | Rohan Takhar |
| University of Liverpool | Ruwanthi Kolamunnage-Dona |
| University of Liverpool | Stephen McKeever |
| University of Manchester | Bernard Keavney |
| University of Manchester | Catriona Harrison |
| University of Manchester | Craig Smith |
| University of Manchester | David Jenkins |
| University of Manchester | Evan Kontopantelis |
| University of Manchester | George Tilston |
| University of Manchester | Glen Martin |
| University of Manchester | Hector Chinoy |
| University of Manchester | Joseph Firth |
| University of Manchester | Lamiece Hassan |
| University of Manchester | Lana Bojanić |
| University of Manchester | Matthew Sperrin |
| University of Manchester | Max Lyon |
| University of Manchester | Maya Buch |
| University of Manchester | Richard Williams |
| University of Manchester | Ruth Norris |
| University of Manchester | Ruth Watkinson |
| University of Manchester | Sarah Steeg |
| University of Manchester | Simon Frain |
| University of Manchester | Simon Williams |
| University of Newcastle | Camille Carroll |
| University of Newcastle | Charlotte Parbery-Clark |
| University of Newcastle | Dexter Canoy |
| University of Newcastle | Precious Onyeachu |
| University of Nottingham | Fiona Pearce |
| University of Nottingham | Laila Tata |
| University of Nottingham | Ralph Akyea |
| University of Nottingham | Stephanie Lax |
| University of Oxford | Aashna Uppal |
| University of Oxford | Akshay Shah |
| University of Oxford | Antonella Delmestri |
| University of Oxford | Antony Palmer |
| University of Oxford | Ben Goldacre |
| University of Oxford | Ben Lacey |
| University of Oxford | Dani Prieto-Alhambra |
| University of Oxford | Eva Morris |
| University of Oxford | George Nicholson |
| University of Oxford | Hayley Evans |
| University of Oxford | James Sheppard |
| University of Oxford | Julia Hippisley-Cox |
| University of Oxford | Kazem Rahimi |
| University of Oxford | Linxin Li |
| University of Oxford | Lucy Wright |
| University of Oxford | Mark Ashworth |
| University of Oxford | Marta Pineda Moncusi |
| University of Oxford | Mohammad Mamouei |
| University of Oxford | Nick Hall |
| University of Oxford | Parag Gajendragadkar |
| University of Oxford | Raph Goldacre |
| University of Oxford | Salma Chaudhry |
| University of Oxford | Sara Khalid |
| University of Oxford | Seb Bacon |
| University of Oxford | Seyed Alireza Hasheminasab |
| University of Oxford | Shishir Rao |
| University of Oxford | Xiaomin Zhong |
| University of Oxford | Zeinab Bidel Taleshmekaeil |
| University of Oxford/KU Leuven | Nathalie Conrad |
| University of Plymouth | Marie-Louise Zeissler |
| University of Sheffield | Jen-Yu Amy Chang |
| University of Sheffield | Norman Briffa |
| University of Sheffield | Peter Bath |
| University of Sheffield | Simone Croft |
| University of Sheffield | Suzanne Mason |
| University of Sheffield | Tim Chico |
| University of Southampton | Nazrul Islam |
| University of Strathclyde | Amanj Kurdi |
| University of Strathclyde | Kim Kavanagh |
| University of Strathclyde | Marion Bennie |
| University of Strathlcyde | Tanja Mueller |
| University of Warwick | Harry Wilde |
| University of Warwick | Majel McGranahan |
| University of Warwick | Sebastian Vollmer |
| University of York | Christina van der Feltz-Cornelis |
| University of York | Han-I Wang |
| University of York | Lorna Fraser |
| Wellcome Sanger Institute | Tapiwa Tungamirai |
| Wellcome Trust | Bilal Mateen |
